# On the Treatment of Lupus Vulgaris

**Published:** 1909-03-27

**Authors:** 


### Dermatology.

### ON THE TREATMENT OF LUPUS VULGARIS.

There are two very important points in regard to the treatment of lupus vulgaris which should never be forgotten. The first of these is that lupus vulgaris should be diagnosed at an early stage and radically treated while the lesions are still small. The second is necessity for continuous treatment. It is mainly owing to failure to recognise lupus while in an early stage, and to the employment of inadequate treatment at this time, that the widely-spread, disfiguring, and often destructive lesions of advanced lupus are so common. With every delay in the radical treatment of early lupus the difficulties of subsequent management and the hope of ultimate cure are diminished. The second important point, the need for continuous treatment, applies more especially to those cases which have been allowed to drift beyond an early stage. It is most essential that the patient be not lost sight of until it is quite certain that no trace of the disease remains. Whatever form of treatment be adopted, careful watch must be kept for recurrences, and any return of the disease must at once be dealt with. Generally, this constant surveillance must be maintained over a number of years.

The methods employed in the treatment of lupus are numerous, and only those which experience has taught to be really trustworthy will be dealt with here. These may be arranged as follows, very much in the order of their merit: (1) excision; (2) Finsen light; (3) *x*-rays; (4) chemical caustics, preceded or not by scraping.

These methods are not to be employed indiscriminately; each is applicable to particular types of lesion only, or to certain stages of the disease.

1. For small lesions, and for lesions up to the size of a crown-piece, when suitably situated, excision is the most certain and satisfactory method. An exception may be made for such lesions upon the face, when the Finsen light is to be employed, if available. Early excision is especially indicated in those now well-known cases of multiple lupus sometimes occurring in children after measles. The incisions should be made well wide of the lesion and should include the subcutaneous fat. In the case of small lesions the wound may be brought together by stitches. With larger lesions skin-grafting should be employed. A few surgeons with special experience are accustomed to remove even very large patches of lupus, and to treat the wound subsequently with skin-grafts and by means of plastic operation; but there are few who are in a position to acquire this experience, and generally other means must be employed for large areas of disease.

2. The method of light treatment introduced by Finsen has now fully established itself as worthy to hold a high place in the treatment of lupus. When properly carried out the results are brilliant and lasting, and for cosmetic purposes this method cannot be excelled, the scar resulting being smooth, fine, and supple. The drawbacks to the treatment are the need of expensive apparatus and of trained nurses, and the length of time required for the cure of any but small areas. There are, however, now many institutions where this treatment is carried out and where even cases of lupus of very wide extent are cured. The treatment is specially valuable for lupus of the face, on account of the absence of deformity by ugly scars which the older treatments by caustics are liable to produce. The Finsen light cannot be employed for lupus on mucous membranes, nor for ulcerating lesions until these have been healed by some other means.

3. At one time it was hoped that in the *x*-rays would be found a solution of all difficulties in the treatment of lupus. Now, although it still remains a most valuable remedy in certain cases, its use is found to be more limited. *X*-rays are especially useful in ulcerated or crusted lupus and in warty forms of lupus. Under treatment by properly measured doses ulcerations heal and crusts fall off, and a so-called "catarrhal lupus" is reduced to its simpler elements—*i.e.* to lupus nodules. At this stage some other form of treatment must be employed, such as Finsen light or caustic applications, for it is not found possible to remove actual nodules of lupus without replacing them by an *x*-ray burn or scar, a proceeding which is never justifiable. For this reason simple non-ulcerated lupus is not suitable for treatment by the rays. On the other hand, in the case of verrucose lupus every trace of the lesion can be removed by *x*-rays without going so far as to produce *x*-ray dermatitis. *X*-ray applications are also very valuable in lupus of mucous membranes.

4. Caustics can never be regarded as an ideal means of treating lupus, but very often they may be the only means at our disposal. They are to be employed when excision is not practicable, when the Finsen light is not available, or the lesions are too extensive for Finsen-light treatment, or for extensive areas upon the limbs where cosmetic results need not be considered. The caustic applications which have from time to time been recommended, and the different methods of applying them, are numerous and varied. Among the drugs employed are caustic potash, arsenic, acid nitrate of mercury, zinc chloride, hydrochloric acid, nitric acid, carbolic acid, salicylic acid, pyrogallic acid, lactic acid, and potassium permanganate. These caustics may be employed in several ways. They may be used alone, or as an adjunct to scraping, or as a preliminary treatment to the use of *x*-rays.

Caustic potash is an old remedy which is still employed by many. It has the advantage over some other caustic applications that its immediate application is not very painful, and that it does not cause lasting after-pain. It may be applied either in stick, the pointed stick being bored into each lupus nodule, or in a 50-per-cent. solution applied and worked in with a match stick. The nodules are thus destroyed, and raw cavities are left which crust over and heal in a few weeks. After an interval the application is repeated until no fresh nodules appear. Pyrogallic acid is applied as a 10-per-cent. ointment in vaseline, and renewed daily as long as the patient can bear the pain and discomfort. The nodules are shelled out as with caustic potash. The affected areas may be frozen and pure hydrochloric acid rubbed over the surface until the nodules stand out white. A second application is made after an interval of some weeks, and so on. Salicylic acid is one of the most useful caustics in lupus, and produces fairly good cosmetic results; it may be employed either as an ointment or in the form of plaster-mull, in both instances with 20 per cent. salicylic acid and 20 per cent. creosote (to deaden the pain of the caustic). The application is changed daily until all nodules have shelled out, and healing is then allowed to take place. None of these caustic methods is, however, entirely satisfactory, for, except in rare superficial lesions, recurrence is the rule. To get the best results it is necessary with this, as with all other forms of treatment, to be on the constant look-out for fresh nodules, and to treat them immediately. For isolated nodules appearing in the scar the actual cautery may be employed, but it should not be used over any large surface on account of the ugly scars which it is likely to leave behind.

Scraping alone has long been abandoned in the treatment of lupus, but for cases beyond the reach of excision, of Finsen light or of $x$-rays, it still remains the best method if followed by the thorough application of caustics. Those which give the best results are caustic potash, acid nitrate of mercury, and salicylic acid. After thoroughly scraping the affected area with the sharp spoon under an anæsthetic, the raw surface is treated with caustic potash (50 per cent. solution) or with the liquid acid nitrate of mercury (B.P.), and dressed with a zinc ointment; or an ointment containing 20 per cent. salicylic acid is immediately applied to the wound, and changed daily until healing takes place. The scars left by scraping and caustics are very apt to become thickened and irregular after a time, and there is a great tendency for fresh nodules to appear.

There still remains one method of treatment in lupus to which it is necessary to refer, namely, that by tuberculin injections. Of this method it may be said that, although there are some who are enthusiastic as to its value in lupus, most of those who have tried it have been disappointed in the results. Ulcerating, crusted, or vegetating cases may improve under this treatment, but it seems to have little or no effect upon unbroken lupus nodules—that is to say, it carries us just to the point which we can reach by other means, but fails where we most want its aid. The same remarks apply almost exactly to the somewhat older method of treatment by thyroid extract.

